# Cephalhematoma and petechial rashes associated with acute parvovirus B19 infection: a case report

**DOI:** 10.1186/1471-2334-13-465

**Published:** 2013-10-07

**Authors:** Masato Takeuchi, Ryosuke Shiozawa, Mayumi Hangai, Junko Takita, Sachiko Kitanaka

**Affiliations:** 1 Department of Pediatrics, The University of Tokyo, Tokyo, Japan; 2 Cell Therapy and Transplantation Medicine, The University of Tokyo, Tokyo, Japan

**Keywords:** Parvovirus B19 infection, Erythema infectiosum, Fifth disease, Papular–purpuric gloves and socks syndrome, Cephalhematoma

## Abstract

**Background:**

Parvovirus B19 can cause petechial rashes in the acute phase of illness as well as erythema infectiosum (fifth disease) during convalescence. This petechial rash is often called “gloves and socks” syndrome because of the typical distribution of the eruption. However, involvement of other sites (e.g., intertriginous area) and generalized involvement have been recently recognized. We report here a patient with parvovirus-associated petechiae and cephalhematoma.

**Case presentation:**

The patient was a previously healthy 10-year-old boy. There was a family history of fatal bleeding; his sister died of intracranial bleeding with an uncertain cause at the age of 5 months. The patient was admitted to our hospital because of sudden onset of cephalhematoma associated with fever. He reported that he had no recent head trauma but that he massaged his scalp on the day before admission. On admission, his temperature was 38.8°C; otherwise, he was in a stable condition. Besides cephalhematoma, petechial rashes were present on his trunk and limbs. The initial laboratory tests were essentially normal, including platelet count and coagulation tests. Expanded laboratory tests were repeated to explore the etiology of his skin hemorrhage, all of which indicated that hematological disorders were unlikely. His symptoms subsided spontaneously over the next few days and he was discharged uneventfully. Anti-parvovirus IgM titer was elevated during hospitalization and typical erythema infectiosum was seen approximately 1 week after discharge. During 6 months follow-up, he remained stable without recurrence of a hemorrhagic episode. Finally, we concluded that his cephalhematoma was responsible for acute parvoviral infection.

**Conclusions:**

This is believed to be the first report describing a possible association between parvovirus B19 infection and cephalhematoma. Parvovirus B19 infection should be considered in the differential diagnosis of children who present with unexplained hemorrhage such as cephalhematoma or petechiae.

## Background

Parvovirus B19 is a causative agent of fifth disease, characterized by facial flushing and diffuse erythema on the proximal extremities in a lacy reticular pattern [1]. This virus also causes arthropathy, aplastic anemia, hepatitis, and fetal infection [1]. In addition to these well-defined manifestations, there is an increasing awareness that parvovirus B19 causes petechial rashes in the viremic phase of illness, which is often termed papular–purpuric gloves and socks syndrome (PPGSS) [2,3]. Recently, petechiae involving other sites have also been recognized [4-6].

Here, we report a case of cephalhematoma and petechial rashes secondary to acute parvovirus B19 infection, as an atypical presentation of PPGSS.

## Case presentation

The patient was a 10-year-old boy. His past medical history was unremarkable, except for premature birth. There was a family history of fatal bleeding, with his sister dying of unexpected intracranial bleeding at the age of 5 months. Her serum protein induced by vitamin K absence-II was markedly elevated on admission; therefore, hemorrhage due to vitamin K deficiency during infancy was suspected. However, she received three doses of oral vitamin K during the neonatal period, which virtually prevented vitamin K deficiency, and she had no known hepatic diseases. The cause of her intracranial bleeding could not be confirmed. Other family members were reportedly healthy without bleeding episodes.

The patient was referred to our hospital because of sudden onset of cephalhematoma associated with fever. The patient and his family reported that he had no recent head trauma but that he massaged his scalp on the day before admission. He was admitted to our hospital for an acute bleeding episode.

**Figure 1:**
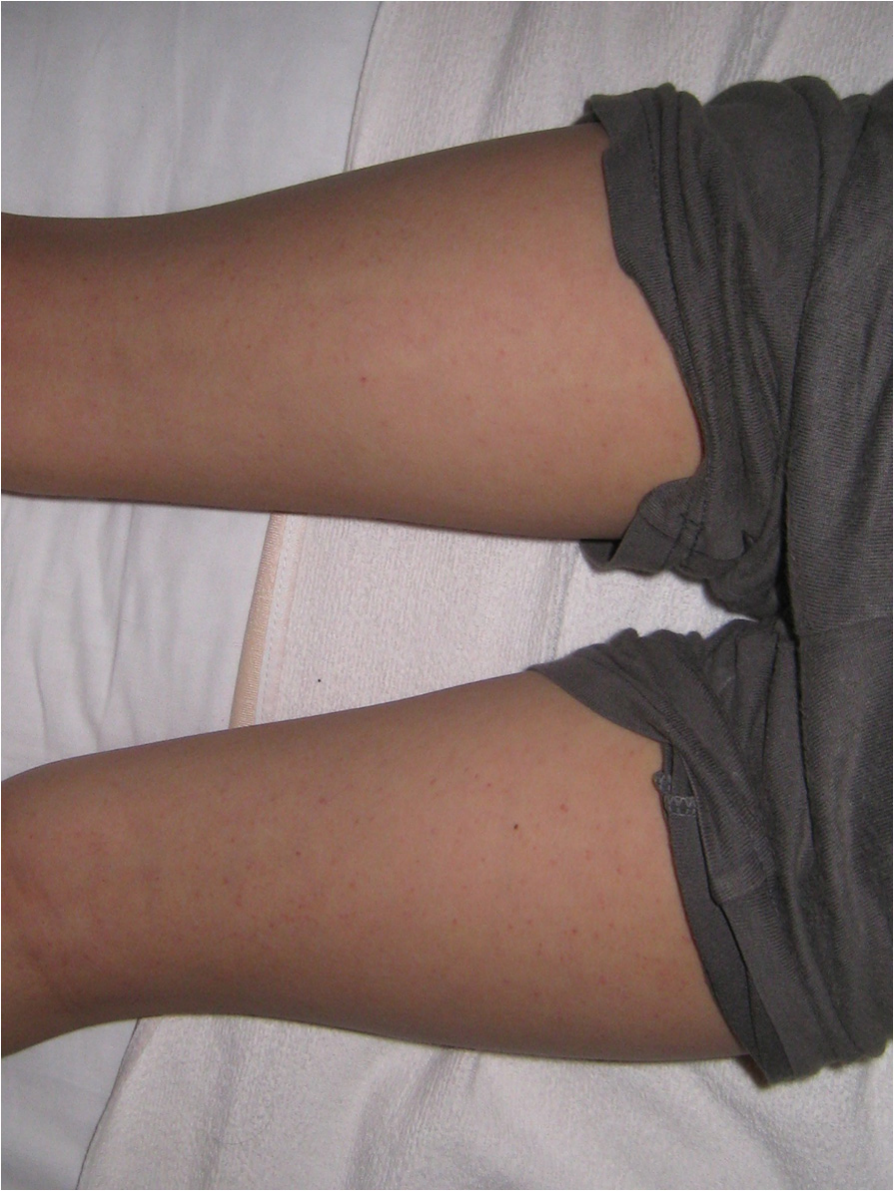
Petechial rashes observed on patient’s legs at admission.

**Figure 2:**
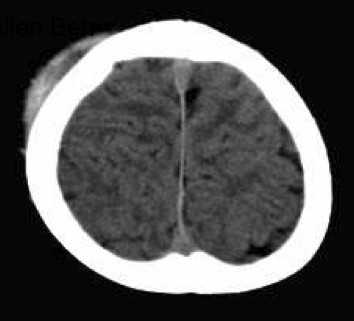
Head computed tomography, revealing subcutaneous hemorrhage without fracture or intracranial bleeding.

**Figure 3:**
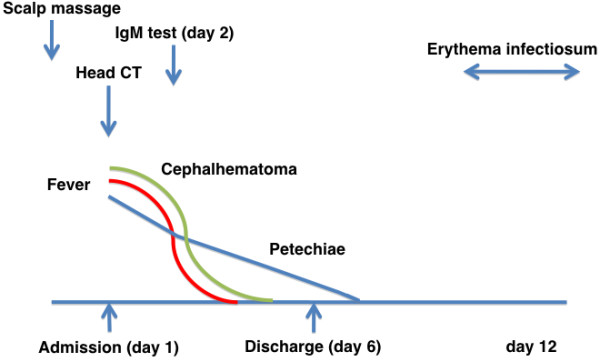
**Clinical course of the present case.** CT: computed tomography.

On admission, his temperature was 38.8°C, but he was in a stable condition. A 5×5-cm^2^ mass was located on the right temporal region of his scalp. The mass was soft and non-tender without signs of inflammation. Petechial rashes were also observed on his oral cavity, trunk, and limbs (Figure 1). At the time of blood sampling, new petechiae easily developed at the tourniquet-compressed site. Table 1 shows the results of laboratory tests. On admission, platelet count and coagulation tests were normal, and mildly impaired white blood cell count and elevated C-reactive protein suggested the presence of a viral infection as the cause of fever. Computed tomography of the head showed subcutaneous hemorrhage without evidence of skull fracture or intracranial bleeding (Figure 2). The presumptive diagnosis on admission was Henoch–Schönlein purpura, although the rashes were non-palpable and the distribution was atypical. Further laboratory tests, including bleeding time, protein C/S, and screening tests for collagen diseases, were repeated to determine the etiology of his skin hemorrhage; all of which indicated that hematological disorders were unlikely in this patient (Table 1). There was no more recognizable bleeding after admission. His symptoms spontaneously subsided over the next few days and he was discharged uneventfully on day 6 of the hospital course (Figure 3).

**Table 1:** **Laboratory data**^**a**^

**Variable**	**Reference range**^**b**^	**Days of hospitalization**				**After discharge**
		**Day 1**	**Day 2**	**Day 3**	**Day 5**	**Day 25**
White cell count (/mm^3^)	3500–9200	2900	2400	2600	3400	4900
Differential count (%)						
Band			7	3	1	1
Segment			54	32	37	37.5
Lymphocytes^c^			28	54	56	54.5
Monocyte			11	8	5	2
Hemoglobin (g/dl)	13.8–16.6	13.1	12.6	13.0	12.2	14.2
Hematocrit (%)	40.2–49.4	38.2	36.0	34.9	35.1	41.4
Reticulocyte (%)	0.8–2.0		0.2	0.1	0.2	1.1
Platelet count (/mm^3^)	150,000–365,000	141,000	111,000	106,000	106,000	151,000
Erythrocyte sedimentation rate (mm/h)	0–7		14			
C-reactive protein (mg/dl)	<0.3	3.26	1.18	0.52	0.12	0.02
Lactate dehydrogenase (U/L)	125–237	214	210	185	202	259
Aspartate aminotransferase (U/L)	9–38	17	18	18	31	18
Alanine aminotransferase (U/L)	4–36	3	3	3	9	3
Total bilirubin (mg/dl)	0.3–1.3	0.8	0.4	0.3	0.4	0.5
Immunoglobulin G (mg/dl)				1130		
Antinuclear antibody (Index)	<40		<40			
Prothrombin time (s)		14.6	11.6	13.1	12.2	11.1
International normalized ratio		1.25	0.99	1.11	1.04	0.97
Activated partial thromboplastin time (s)	25.5–36.1	30.2	28.8	28.6	28.2	30.5
Protein induced by vitamin K absence-II (mAU/ml)	<40		32			
Fibrinogen (mg/dl)	186–355	306	309	266	242	239
D-dimmer (μg/dl)	<2.5		<0.5			
Antithrombin (%)	83–128		101			
Thrombin–antithrombin complex (ng/ml)	0–2.9		1.6			
Protein C (%)	75–154		69			
Protein S (%)	74–132		71.2			
Platelet aggregation activity				Normal		
Bleeding time	<5 min		2 min and 30 s			
Parvovirus B19 IgM (Index)	<0.8			10.06		
EBV-VCA IgM (Index)^4^	<10		10			
EBV-VCA IgG (Index)^4^	<10		160			
EBNA	<10		40			

^a^Because this patient was admitted during weekend, the available tests were reduced on day 1 (e.g., differential count on smear).

^b^Reference range at our institution, not adjusted for age. Blank column indicates that reference range was not available.

^c^Atypical lymphocytes were not observed.

^4^measured by fluorescent antibody technique.

EB, Epstein–Barr virus; EBNA, Epstein–Barr nuclear antigen; VCA, viral capsid antigen.

Parvovirus-B19-specific IgM tested during hospitalization, which was measured using the Parvo IgM-EIA “SEIKEN” kit (Denka Seiken Inc, Tokyo, Japan), showed a positive result (Table 1) and typical erythema infectiosum was observed approximately 1 week after discharge. The diagnosis of acute parvovirus infection on admission was established based on clinical grounds as well as serological evidence. During 6 months of follow-up, he remained well without recurrence of a hemorrhagic episode. Finally, we concluded that cephalhematoma was the result of acute parvoviral infection.

## Discussion

Parvovirus B19 is associated with a variety of symptoms, involving skin, joint and hematological manifestations [1]. Among the cutaneous manifestations, PPGSS was first described by Harms et al. in 1990 [7]_._ Bagot et al. reported in 1991 that PPGSS might be related to primary infection with parvovirus B19 [8]. Since then, there have been a growing number of publications supporting the relationship between PPGSS and acute parvovirus infection [2,3]. For example, 13 cases with parvovirus-associated purpura were reported during a community outbreak [5]. Initial case reports and case series documented that the eruptions were limited to the hands and feet. In contrast, according to recent studies, rashes are also present at other sites, such as the face, trunk, and the intertriginous area, or they may be generalized [4-6]. Based on these observations, the term “parvovirus B19-associated purpuric–petechial eruption” was proposed because PPGSS may not represent the characteristics of these types of eruptions [6]. Although the pathogenesis of petechial rashes has not been established, there is some evidence showing that direct skin invasions of parvovirus B19 could be a cause [9].

Our patient was hospitalized during the viremic period of parvovirus B19 infection, as shown by the presence of fever, elevated IgM antibody, and subsequent erythema infectiosum. Accordingly, petechial rashes in this patient were consistent with those seen in acute parvoviral infection, and were well defined. Additionally, we believe that cephalhematoma resulted from minor external pressure (scalp massage) during the viremic phase of parvovirus infection. This belief was supported by the lack of hematological disorders explaining the hemorrhagic events. In addition, the nature of the hemorrhage was consistent with previous observations that parvovirus-associated petechiae are seen in intertriginous areas. Differential diagnoses included Gianotti–Crosti syndrome, hand, foot and mouth disease, erythema multiforme, Henoch–Schönlein purpura, and Kawasaki disease [3]. However, such diseases could not sufficiently explain the clinical course (e.g., features of eruption or lack of associated symptoms) and cephalhematoma has not been reported as a complication of these conditions. Therefore, we believe that acute parvoviral infection was the most likely explanation for the cephalhematoma and petechial rashes in our patient. If this is correct, to the best of our knowledge, this is the first report of an association between acute parvovirus infection and cephalhematoma that is not attributable to trauma or hematological abnormalities.

This case report had two potential limitations: interpretation of the serological data and involvement of other viruses. There is controversy over the serological diagnosis of acute parvovirus B19 infection. Some specialists emphasize that IgM measurement alone is sufficient [10,11], whereas others argue that combination of IgG/IgM and/or DNA detection is essential [12]. In our case, we relied solely on parvovirus IgM for the serological diagnosis of acute infection and seroconversion of IgG was not confirmed. This was attributed to the medical insurance system in Japan, which does not cover measurement of parvovirus IgG; out-of-pocket expenditure is required to test for this antibody. The lack of other laboratory evidence may lead to misclassification of the disease, such as a false-positive result [13]. However, regarding the performance of IgM measurement, Doyle et al. reported specificity of 99.4% with excellent reproducibility when the cut-off index of 0.8 was applied [14]. Another study found that, in 83 samples during the viremic phase of parvovirus B19 infection, none had false-positive IgM [15]. According to these studies, the possibility of false-positive IgM is <1% and we believe that IgM of 10.06 in our case was sufficient to diagnose acute parvoviral infection. Second, sporadic association between PPGSS and other viruses has been also reported, including varicella–zoster virus, Epstein–Barr virus (EBV), cytomegalovirus, herpesvirus 6/7, Coxsackie virus, hepatitis B virus, and rubella virus [3]. Moreover, co-infection with herpesvirus 6 and parvovirus was also reported in a case with PPGSS [2]. Among those viruses, we examined only EBV, which showed no evidence of acute infection. Therefore, we did not fully screen potentially relevant viruses other than EBV and parvovirus. This may be another limitation of our report.

## Conclusions

We describe a patient with cephalhematoma and petechial rashes accompanied by acute parvovirus B19 infection. Patients in the viremic phase of parvoviral infection may manifest subcutaneous bleeding such as cephalhematoma. Parvovirus infection should be considered in the differential diagnosis of patients who present with unexplained hemorrhage, including cephalhematoma and petechial eruptions.

## Consent

Written informed consent was obtained from both the patient and his guardian for publication of this Case Report and any accompanying images. A copy of the written consent is available for review by the Editor of this journal.

## Abbreviations

EBV: Epstein–Barr virus; PPGSS: Papular–purpuric gloves and socks syndrome.

## Competing interests

The authors declared that they have no competing interests.

## Authors’ contributions

All authors were involved in the discussion of the present case, including the evaluation and management. MT had the main responsibility for preparing the first draft of the manuscript. All authors read and approved the final draft.

## Pre-publication history

The pre-publication history for this paper can be accessed here:

http://www.biomedcentral.com/1471-2334/13/465/prepub
